# Lymphocyte DNA methylation mediates genetic risk at shared immune-mediated disease loci

**DOI:** 10.1016/j.jaci.2019.12.910

**Published:** 2020-05

**Authors:** Alexander D. Clark, Nisha Nair, Amy E. Anderson, Nishanthi Thalayasingam, Najib Naamane, Andrew J. Skelton, Julie Diboll, Anne Barton, Stephen Eyre, John D. Isaacs, Arthur G. Pratt, Louise N. Reynard

**Affiliations:** a Newcastle University Translational and Clinical Research Institute, Newcastle upon Tyne, United Kingdom; b Versus Arthritis Centre for Genetics and Genomics, Centre for Musculoskeletal Research, Institute of Inflammation and Repair, University of Manchester Manchester, United Kingdom; c NIHR Manchester Musculoskeletal BRC, Manchester University NHS Foundation Trust, Manchester, United Kingdom; d Bioinformatics Support Unit, Faculty of Medical Sciences, Newcastle University, Newcastle upon Tyne, United Kingdom; e Musculoskeletal Services Directorate, Newcastle upon Tyne Hospitals NHS Trust, Newcastle upon Tyne, United Kingdom; f Newcastle University Biosciences Institute, Newcastle upon Tyne, United Kingdom

**Keywords:** Rheumatoid arthritis, adaptive immunity, immune-mediated disease, CD4^+^ T cell, B cell, genetics, expression quantitative trait locus, DNA methylation, methylation quantitative trait locus, pathogenesis, CIT, Causal inference test, DNAm, DNA methylation, eQTL, Expression quantitative trait locus, eQTM, Expression quantitative trait methylation, FDR, False-discovery rate, GWAS, Genome-wide association study, IMD, Immune-mediated disease, LD, Linkage disequilibrium, meQTL, Methylation quantitative trait locus, MS, Multiple sclerosis, OA, Osteoarthritis, RA, Rheumatoid arthritis, SNP, Single nucleotide polymorphism, TFBS, Transcription factor binding site

## Abstract

**Background:**

Defining regulatory mechanisms through which noncoding risk variants influence the cell-mediated pathogenesis of immune-mediated disease (IMD) has emerged as a priority in the post–genome-wide association study era.

**Objectives:**

With a focus on rheumatoid arthritis, we sought new insight into genetic mechanisms of adaptive immune dysregulation to help prioritize molecular pathways for targeting in this and related immune pathologies.

**Methods:**

Whole-genome methylation and transcriptional data from isolated CD4^+^ T cells and B cells of more than 100 genotyped and phenotyped patients with inflammatory arthritis, all of whom were naive to immunomodulatory treatments, were obtained. Analysis integrated these comprehensive data with genome-wide association study findings across IMDs and other publicly available resources.

**Results:**

We provide strong evidence that disease-associated DNA variants regulate *cis*-CpG methylation in CD4^+^ T and/or B cells at 37% RA loci. Using paired, cell-specific transcriptomic data and causal inference testing, we identify examples where site-specific DNA methylation in turn mediates gene expression, including *FCRL3* in both cell types and *ORMDL3/GSDMB*, *IL6ST/ANKRD55*, and *JAZF1* in CD4^+^ T cells. A number of genes regulated in this way highlight mechanisms common to RA and other IMDs including multiple sclerosis and asthma, in turn distinguishing them from osteoarthritis, a primarily degenerative disease. Finally, we corroborate the observed effects experimentally.

**Conclusions:**

Our observations highlight important mechanisms of genetic risk in RA and the wider context of immune dysregulation. They confirm the utility of DNA methylation profiling as a tool for causal gene prioritization and, potentially, therapeutic targeting in complex IMD.

## Graphical abstract

**Figure:**
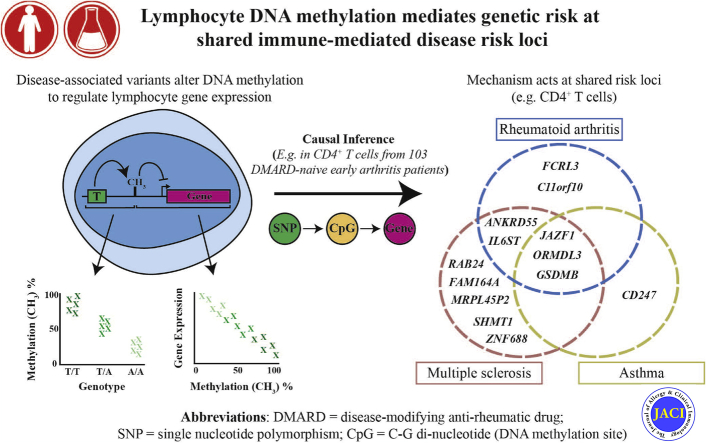

Genome-wide association studies (GWASs) have shed light on the genetic architecture of common immune-mediated diseases (IMDs). However, disease-associated variants are typically noncoding, leaving the mechanism(s) through which they contribute to etiology unclear. For example, to date single nucleotide polymorphisms (SNPs) at more than 100 genomic loci have been found to influence rheumatoid arthritis (RA) susceptibility,^1^ and their enrichment at enhancer elements that are active in CD4^+^ T and B cells suggests that substantial risk is conferred via transcriptional dysregulation of the adaptive immune system.^2^, ^3^, ^4^ By mapping allelic effects on gene expression—termed expression quantitative trait loci (eQTLs)—we and others have sought to prioritize candidate causal genes in these cells.^5^, ^6^, ^7^, ^8^ However, SNPs also modulate DNA methylation (DNAm; *methylation* quantitative trait loci [meQTLs]),^9^, ^10^, ^11^, ^12^, ^13^, ^14^, ^15^ whose role in transcriptional regulation is now well established.^16^ The colocalization of meQTLs with eQTLs therefore implicates DNAm as a potential mediator of observed eQTL effects in some cases,^9^, ^10^, ^11^, ^12^, ^13^, ^14^, ^15^ and intriguing associations between site-specific DNAm and the development of RA have been documented in several cell types.^17^, ^18^, ^19^, ^20^, ^21^, ^22^, ^23^ Identifying instances in which RA-associated SNPs impact DNAm in a manner that affects lymphocyte gene transcription could substantially refine the regulatory landscape of candidate genes in IMDs such as RA.

eQTL effects often differ between cell types^10^^,^^12^^,^^24^ and although deconvolution methods can be applied to mixed-cell populations,^25^ the role of meQTLs in disease pathogenesis should ideally be validated in isolated subsets. Furthermore, DNAm and gene expression may be shaped by the local microenvironment in which a cell exists,^21^ with certain eQTL effects becoming evident only on cell stimulation or in the context of active inflammation,^26^^,^^27^ and site-specific DNAm may be similarly linked to acute-phase response.^28^^,^^29^ Hence, meQTL-mediated mechanisms of genetic risk are optimally evaluated at a cellular level in relevant patient cohorts, and doing so should yield important insight into complex disease pathogenesis. Here, we used genome-wide molecular profiling to comprehensively investigate the relationship between RA-associated genetic variants, DNAm, and gene expression in primary CD4^+^ T and B cells of drug-naive patients with early arthritis. Our findings are interpreted in the context of publicly available data sets.

## Methods

Fully detailed methods are described in this article’s Methods section in the Online Repository at www.jacionline.org.

### Lymphocyte-specific nucleic acid isolation from patients

Patients of Northern European ancestry with suspected inflammatory arthritis were recruited before treatment with immunomodulatory drugs as described.^30^ Patients with RA were classified using current, internationally accepted criteria,^31^ and matched with disease controls in respect of demographic and clinical characteristics. CD4^+^ T cells and CD19^+^ B cells were isolated from fresh peripheral blood using magnetic bead–based positive selection, with purity confirmed by flow cytometry, and DNA/RNA extracted as described.^8^ The study was approved by the Newcastle and North Tyneside Regional Ethics Committee, and all participants gave informed consent.

### Genotyping

Genotyping was carried out using an Illumina Human CoreExome-24 version 1-0 array (Illumina, San Diego, Calif). Samples and SNPs with a call rate of less than 98% were excluded, as were SNPs with a minor allele frequency of less than 0.01 or Illumina GenomeStudio cluster separation of less than 0.4. Data were prephased with SHAPEIT2^32^ and imputed to the 1000 Genomes Phase 3 reference panel using IMPUTE2,^33^ with imputed SNPs having INFO scores of less than 0.8 being removed. Quantitative trait locus analysis was limited to SNPs for which there were 3 or more individuals per genotype or, in the absence of minor allele homozygotes, 8 or more heterozygous individuals.

### DNAm quantification and meQTL analysis

Four hundred nanogram of DNA was bisulphite-converted and DNAm quantified using the Infinium MethylationEPIC BeadChip (Illumina). After independent preprocessing and functional normalization^34^ of CD4^+^ T- and B-cell data, probe filtering was performed and surrogate variable analysis used to estimate confounding variables (surrogate variable analysis package^35^), conserving the effects of disease diagnosis. These were then included as covariates for subsequent meQTL modeling in the MatrixEQTL package.^36^ False-discovery rate (FDR) was calculated across all tests, and independent signals distinguished by SNP clumping. In addition, an interaction analysis (*Genotype × Diagnosis*) was performed to identify putative disease-specific effects. RA-meQTLs were defined where disease risk loci, extracted from publicly available GWAS catalog studies,^37^ mapped to the meQTL regulatory SNP, with Bayesian colocalization applied to provide additional evidence for a shared causal variant.^38^ GWAS variants from studies of multiple sclerosis (MS), asthma, and osteoarthritis (OA) were similarly leveraged to extend analyses beyond RA loci.

### Gene expression quantification

CD4^+^ T- and B-cell transcriptomic data were generated using the HumanHT-12 v4 Expression BeadChip (Illumina) as has been described.^8^ Failed probes were removed before background correction and quantile normalization in Limma.^39^ Additional probes were filtered where they mapped to (1) sex chromosomes, (2) repeat or intronic/intergenic regions, or (3) unmapped regions. Surrogate variable analysis was performed as for DNAm data.^35^

### Chromatin state and transcription factor binding enrichment analysis

*Cis*-CpG sites associated with GWAS loci were mapped to chromatin state information from primary T_H_ cells and primary B cells as defined by the Roadmap Epigenomics consortium,^40^ and similarly with chromatin immunoprecipitation sequencing–derived transcription factor binding site (TFBS) data from the *Encyclopaedia of DNA Elements* project.^41^^,^^42^ Enrichment of risk-associated meQTL CpGs at specific chromatin states and TFBSs was determined with the Fisher exact test, with nonrisk *cis*-CpGs used as background for enrichment analyses. Gene ontology enrichment analysis of biological pathways was performed using the gometh function within the missMethyl package.^43^

### Expression quantitative trait methylations and causal inference

At risk-associated *cis*-CpG sites, Spearman rho (FDR < 0.01) was applied with transcripts within ±500 Kb to identify expression quantitative trait methylations (eQTMs). To infer directionality of SNP-CpG-transcript associations, a causal inference test (CIT) was applied,^44^ inputting triplets at disease risk loci that demonstrated both *cis*-meQTL and *cis*-eQTM effects. FDR values were determined by performing 1000 data permutations.

### Validation of quantitative trait locus effects by pyrosequencing and luciferase reporter assay

*Cis*-meQTL effects at 3 loci implicated by CIT were replicated by pyrosequencing^45^ in an independent cohort of genotyped patients with early arthritis. Analysis of allelic expression imbalance was used to further confirm the eQTL effect at the exemplar *FCRL3* locus in CD4^+^ T cells.^46^ Finally, the inferred DNAm-mediated regulation downstream of the regulatory SNP was assessed experimentally at this locus using a luciferase reporter assay in the Jurkat T-cell line. For methodological detail, see this article’s Methods section in the Online Repository (primer sequences for all pyrosequencing and *FCRL3* promoter amplification are listed in Table E1 in this article’s Online Repository at www.jacionline.org).

### Statistical analysis

Statistical analyses were performed in R (http://www.R-project.org/) version 3.4.4 and GraphPad Prism 7 (San Diego, Calif). A *P* value of less than .05 was considered significant unless otherwise stated.

## Results

### Patient cohort and clinical characteristics

Paired genotype and DNAm data were available from 141 patients with early arthritis (CD4^+^ T cells alone in 22 cases, B cells alone in 38, and both cell types in 81). The demographic and clinical characteristics of donors are presented for each cell type in Table I. Comparator groups within each cohort (RA and non-RA) were matched for major demographic and clinical parameters including age, sex, and markers of systemic inflammation (C-reactive protein; erythrocyte sedimentation rate). The disease control group comprised a range of diagnoses, most being spondyloarthropathies.

**Table I:** Description of patient cohorts included in the meQTL analysis of CD4^+^ T cells and B cells

Characteristic	Patients with RA	Disease controls	*P* value∗
**CD4**^**+**^**T cells**			
No. of patients	43	60	
Age (y)	58 (50-69)	54 (46-63)	.13
Sex: female, %	67	70	.78
C-reactive protein (mg/L)	9 (5-13)	7.5 (5-13.5)	.24
Erythrocyte sedimentation rate (mm/h)	19 (7-32)	15 (9-29)	.74
CCP-positive, %	47	—	
RF-positive, %	58	—	
Tender 28	3 (0-11)	2 (0-5.5)	.39
Swollen 28	1 (0-3)	0 (0-2)	.51
Diagnosis in disease controls, %			
OA	8		
Other noninflammatory arthritis	7		
Spondyloarthropathy (PsA, ReA, EA)	45		
Crystal arthropathy	15		
Other inflammatory arthritis	13		
Other/undifferentiated	12		

**B cells**			
No. of patients	46	73	
Age (y)	57 (50-68)	55 (46-64.5)	.34
Sex: female, %	74	71	.75
C-reactive protein (mg/L)	9 (5-13)	7 (5-14.5)	.14
Erythrocyte sedimentation rate (mm/h)	19.5 (5.75-34.5)	16 (9-30.75)	.99
CCP-positive, %	54	—	
RF-positive, %	61	—	
Tender 28	3 (1-9)	3 (1-6)	.50
Swollen 28	1 (0-3)	0 (0-3)	.52
Diagnosis in disease controls			
OA	8		
Other noninflammatory arthritis	7		
Spondyloarthropathy (PsA, ReA, EA)	48		
Crystal arthropathy	11		
Other inflammatory arthritis	8		
Other/undifferentiated	18		

Statistics presented are median (interquartile range) for continuous data and percentages for discrete data.

∗ *P* values reported are calculated between RA and disease control groups using a χ^2^ test for categorical data, or Mann-Whitney test for continuous data.

### Genome-wide mapping highlights both common and differential *cis*-meQTL effects between CD4^+^ T and B cells in early arthritis

**Fig 1:**
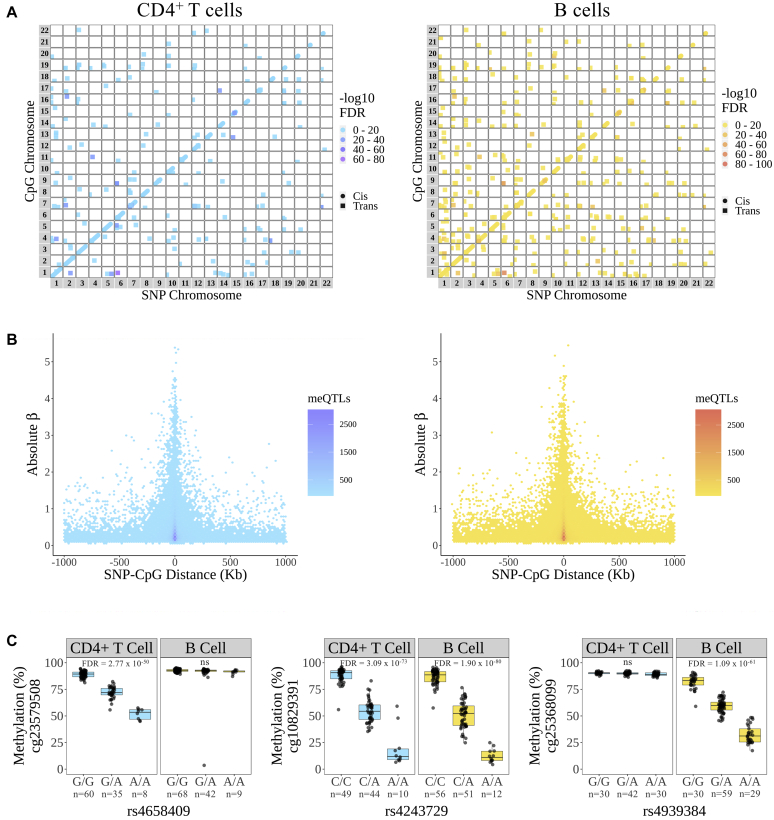
meQTL mapping genome-wide in CD4^+^ and B cells of patients with early arthritis. **A,** SNP and CpG coordinates of all independent *cis*/*trans*-meQTL effects mapped in both CD4^+^ T cells (*left*) and B cells (*right*) across patients with early arthritis. *Cis*-meQTLs denoted by circles and *trans* meQTLs by squares, with the color reflecting the −log_10_ FDR value of the association. **B,** Distance between regulatory SNP and its cognate CpG site plotted against effect size (absolute regression coefficient, *β*) for all *cis*-meQTLs. **C,** Exemplar plots of *cis*-meQTLs identified exclusively CD4^+^ T cells (*left*), common to both cell types (*middle*), or exclusively in B cells (*right*). Boxplots display the median, 25th and 75th percentiles. Whiskers extend to the highest/lowest value that is no greater than 1.5 3 interquartile range from the 75th/25th percentile, respectively.

**Fig E1:**
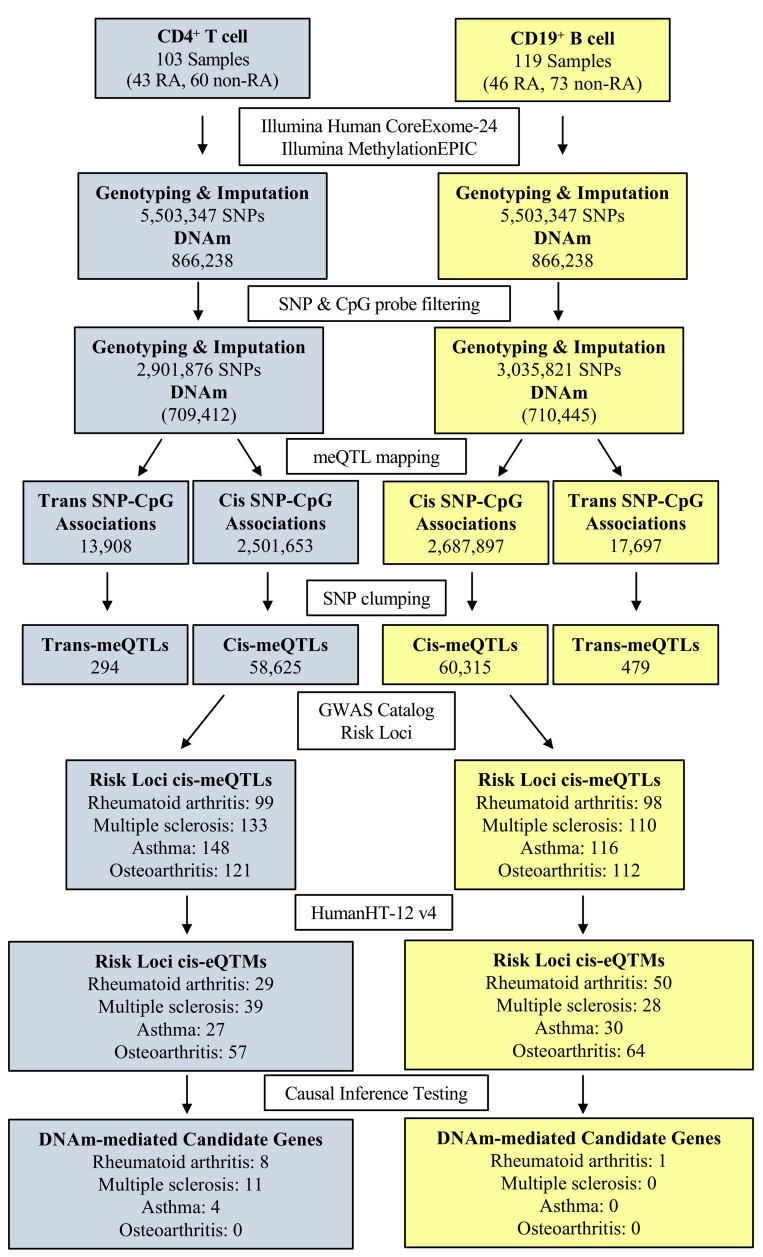


meQTL effects (*cis* and *trans*) were mapped genome-wide in each lymphocyte subset across all patients (Fig 1, *A*; Fig E1 in this article’s Online Repository at www.jacionline.org provides an overview of study design and key findings). We predominantly identified meQTLs acting in *cis*, defined as an SNP-CpG association occurring over a distance of less than 1 megabase (see this article’s Methods section in the Online Repository). Focusing on these, 58,625 independent *cis*-meQTLs were active in CD4^+^ T cells and 60,315 in B cells (Fig 1, *A*; see Tables E2 and E3 in this article’s Online Repository at www.jacionline.org), involving 53,131 and 53,925 individual CpGs, respectively.

**Fig E2:**
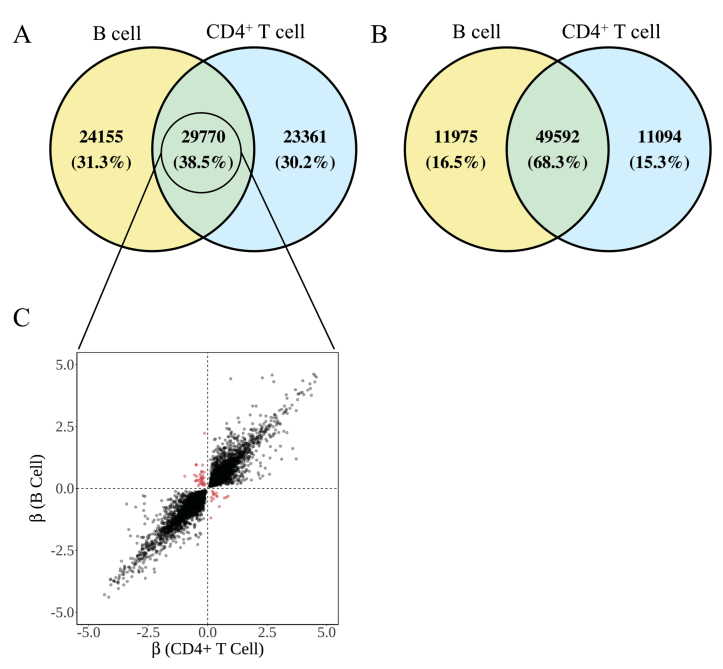


**Fig E3:**
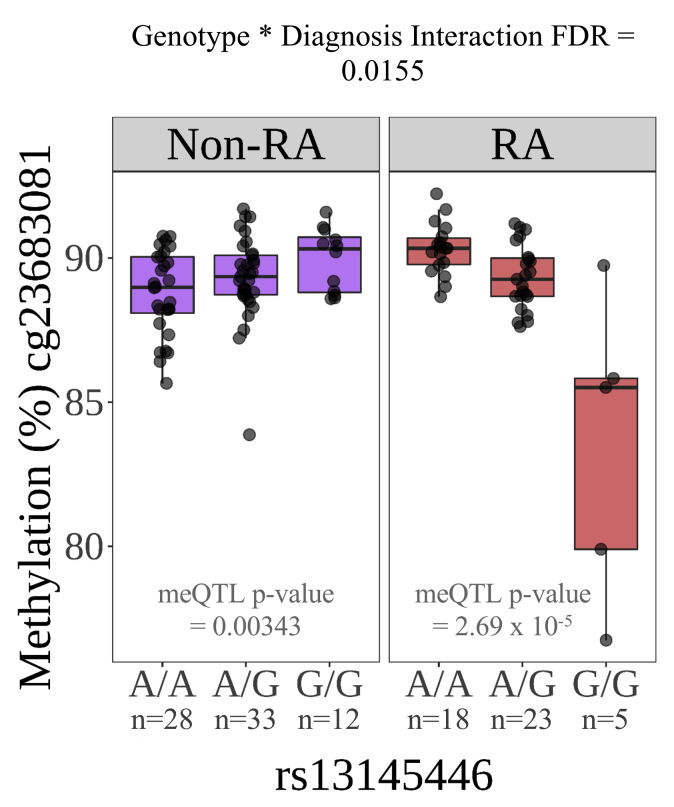


A diminution of meQTL effect size (*β* coefficient) was observed with increasing SNP-CpG distance, consistent with a primarily *cis*-regulatory function of these variants (Fig 1, *B*). Correspondingly, 87% of the observed *cis*-meQTLs in CD4^+^ T cells and 85% in B cells occurred over a distance of less than 250 kb. Exemplar plots of significant SNP-CpG *cis*-regulatory associations that were identified in both cell types, or showed significant associations only in CD4^+^ T cells or B cells, are shown in Fig 1, *C*. A substantial proportion of CpGs subject to *cis*-meQTL effects (*cis*-CpGs) were common to CD4^+^ T and B cells; indeed, a powerful empirical Bayes analytical approach^47^ suggested that as many as 68% of associations in either cell type were shared (see Fig E2, *A* and *B*, in this article’s Online Repository at www.jacionline.org). The direction and effect size of overlapping *cis*-meQTLs were approximately equivalent between CD4^+^ T and B cells, though opposing allelic effects appeared to be exerted at 47 CpGs (Fig E2, *C*, shown in red; see Table E4 in this article’s Online Repository at www.jacionline.org). We also explored the possibility that meQTL effects may be impacted by disease phenotype (RA *vs* non-RA; see this article’s Methods section in the Online Repository). Such effects in *cis* were scarce, with only 1 example occurring in B cells (see Fig E3 and Table E5 in this article’s Online Repository at www.jacionline.org).

### *cis*-CpGs at-risk loci are functionally enriched according to cell type–specific chromatin architecture

**Fig 2:**
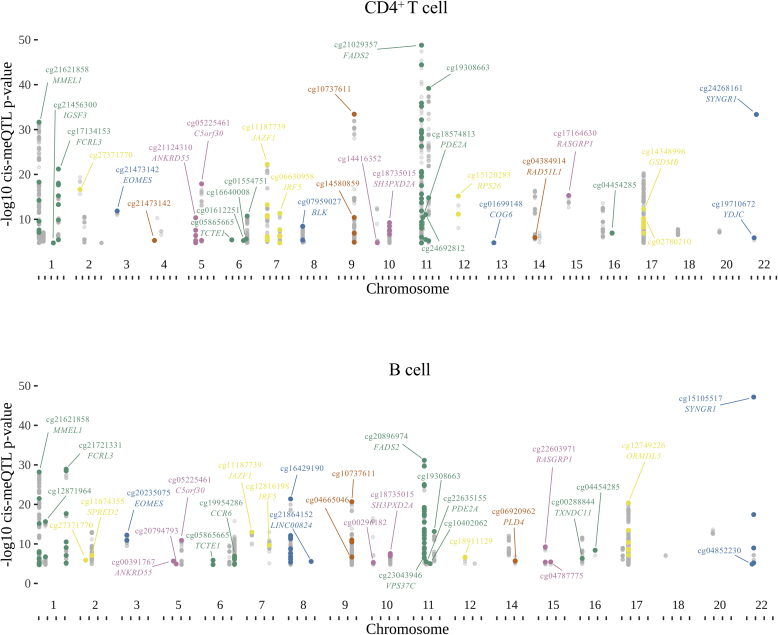
Manhattan plots show significance of *cis* SNP-CpG associations satisfying the genome-wide FDR threshold that maps to LD blocks harboring RA risk variants from the GWAS Catalog.^37^ CD4^+^ T-cell associations are shown in the upper plot, and B-cell associations are shown in the lower plot. SNP-CpG pairs highlighted in color are the top independent associations at each locus after SNP clumping. Gene names are given for instances where CpG sites fall at annotated genes in the Illumina annotation file.

We sought evidence that site-specific DNAm represents a functional intermediary of genetic risk for complex disease, identifying instances in which meQTL regulatory variants map to risk loci from published GWAS data.^37^ First focusing on RA, linkage disequilibrium (LD) blocks harboring risk-associated SNPs from case-control studies were intersected with the meQTL landscape of early arthritis lymphocytes. Of the independent RA risk loci represented in our data sets (104 and 107 for CD4^+^ T and B cells, respectively), 24 exhibited *cis*-regulatory effects on DNAm in both cell types, with 8 identified uniquely in CD4^+^ T cells and 9 in B cells (Fig 2; see Table E6 in this article’s Online Repository at www.jacionline.org). An independent, Bayesian statistical approach provided strong corroborative evidence (PP4 > 0.75; see this article’s Methods section in the Online Repository) that disease- and methylation-associated variants colocalized in most of these cases (76% of RA *cis*-meQTLs in CD4^+^ T cells, 79% in B cells; see Table E6). CpGs identified were typically located within, or proximal to, genes previously proposed to be candidates at RA risk loci^1^ and/or those observed to be the subject of lymphocyte eQTLs described by us and others^5^, ^6^, ^7^, ^8^ (Fig 2).

**Fig 3:**
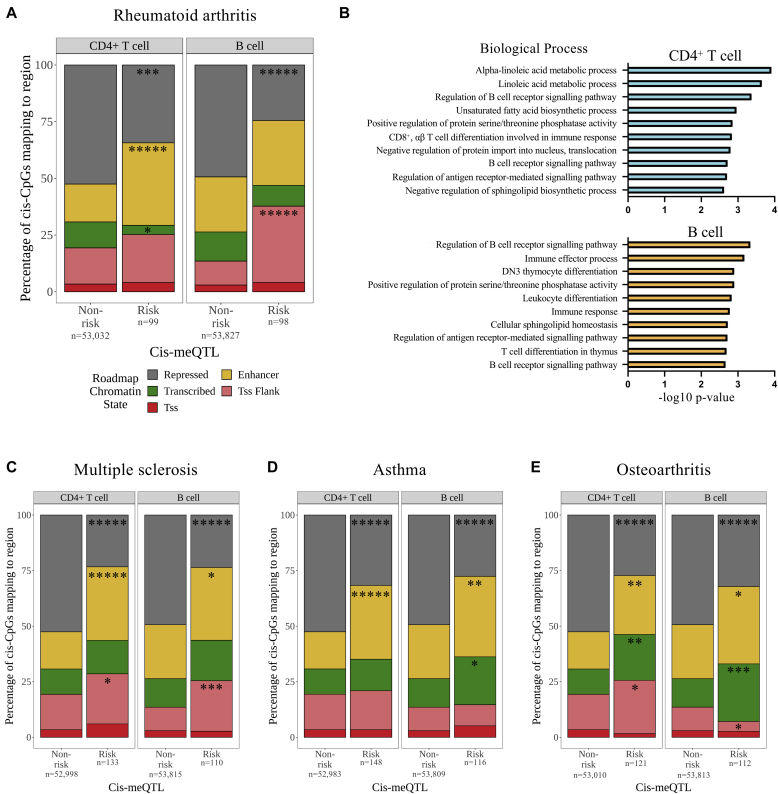
**A,** Relative enrichment of RA risk-associated CpGs at primary T_H_-cell– and B-cell–specific chromatin states defined by the Roadmap Epigenomics project^40^ (15 chromatin states collapsed into indicated 5 groups indicated in key; see this article’s Methods section in the Online Repository). Relative chromatin enrichments were calculated using the Fisher exact test, *P < .05; **P < .01; ***P < .001; ****P < .0001; *****P < .00001. **B,** Top 10 biological gene ontology processes enriched at risk CpG-associated genes in T cells (*top*) and B cells (*bottom*). Relative enrichment of MS (**C**), asthma (**D**), and OA (**E**) risk-associated CpGs at primary T_H_-cell– and B-cell–specific chromatin states, analogous to Fig 3, *A*.

To understand better the regulatory effects of disease-associated loci, we mapped *cis*-CpGs genome-wide to cell-specific chromatin states as described by the Roadmap Epigenomics Project.^40^ At RA risk loci in CD4^+^ T cells, such CpGs were enriched at enhancer elements and depleted at transcribed and repressed regions, relative to those associated with non-risk loci (Fig 3, *A*). Analogous enrichment was observed at transcription start site–flanking regions in B cells, again with underrepresentation at sites of repressed chromatin (Fig 3, *A*). Because DNAm can influence protein-DNA interactions,^48^ meQTLs could confer disease risk via altered transcription factor binding. Consistent with this, by mapping meQTL-associated CpGs to TFBSs determined from publicly available data,^41^^,^^42^ we found that those at RA risk loci of CD4^+^ T and B cells were overrepresented at nuclear factor kappa B subunit p65-binding sites (see Table E7 in this article’s Online Repository at www.jacionline.org). Gene ontology analysis revealed processes relating to immune cell function/development to be enriched at RA meQTL *cis*-CpGs of both cell types, compared with those outside risk loci (Fig 3, *B*; see Table E8 in this article’s Online Repository at www.jacionline.org). Prominent among these were *regulation of the B-cell receptor signaling pathway* and *regulation of antigen receptor–mediated signaling pathway*.

Twenty of 41 (49%) RA-associated *cis*-meQTLs identified were noted to reside at risk loci shared with other IMDs (Table E6). Focusing on asthma and MS, we therefore explored whether *cis*-meQTLs at risk loci mapped similarly to cell-specific chromatin states in such diseases, and contrasted this with OA, an arthropathy in which dysregulated adaptive immunity is considered a comparatively minor contributor to pathogenesis. Across all traits, risk-associated CpG sites were depleted in repressed chromatin regions relative to nonrisk CpGs. However, we observed a particularly marked enrichment of IMD-associated CpG sites in CD4^+^ T-cell enhancers (≥2-fold compared with nonrisk CpGs; see Fig 3, *A* and *C*, *left panels*). *Cis*-CpGs associated with OA risk loci were less enriched in lymphocyte regulatory elements (eg, 1.6-fold in CD4^+^ T-cell enhancers) but were, in contrast, overrepresented in transcribed regions (Fig 3, *C*, *right panel*). MS-associated *cis*-CpGs (though not asthma-associated equivalents) were enriched at *RELA*, *BATF*, and *RUNX3* TFBSs (see Tables E9 and E10 in this article’s Online Repository at www.jacionline.org). Ontology enrichment analyses of both IMD-associated meQTL landscapes implicated lymphocyte activation/differentiation, in addition to cytokine and type 2 immune responses at B-cell meQTLs in asthma (see Tables E11 and E12 in this article’s Online Repository at www.jacionline.org). Conversely, the OA-associated meQTL landscape highlighted a distinct TFBS profile (see Table E13 in this article’s Online Repository at www.jacionline.org), and pathways related to cellular apoptosis and endopeptidase activity (see Table E14 in this article’s Online Repository at www.jacionline.org). Taken together, these data point to a role for DNAm in modulating functionally relevant transcriptional activity of lymphocyte subsets that are most prominent at shared IMD risk loci.

### DNAm mediates eQTL effects at RA risk loci

**Fig 4:**
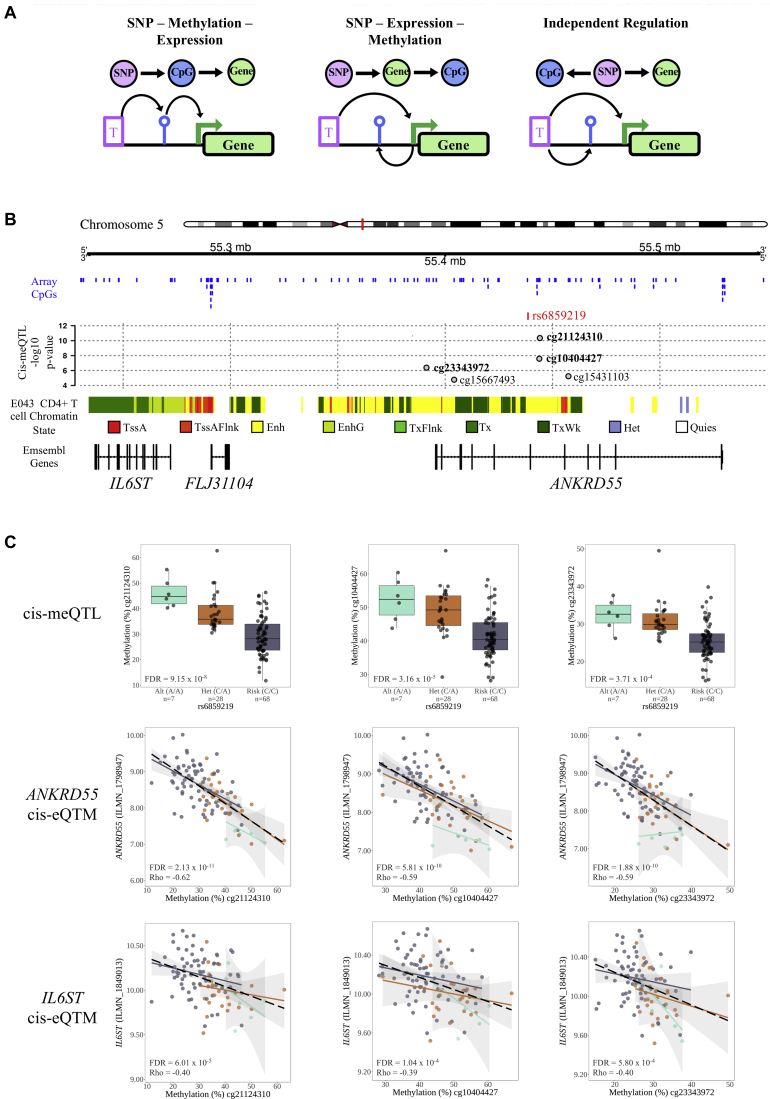
DNAm-mediated transcriptomic regulation of *ANKRD55* and *IL6ST* at the risk locus on chromosome 5q11.2 in CD4^+^ T cells. **A,** Potential regulatory models explaining the association between genotype at-risk variants and associations with both DNAm status at CpGs in *cis* and transcriptional activity at proximal genes. **B,** The RA risk variant rs6859219 (*red line*) at chromosome 5q11.2 is associated with DNAm at 4 intronic and 1 intergenic (cg23343972) CpG sites (gray circles), mapping to CD4^+^ T-cell enhancer elements (*yellow*). Blue lines indicate the position of all CpG sites included in the meQTL analysis. **C,** meQTL (*top*) and eQTM plots for 3 of the 5 CpG sites associated with the rs6859291 risk variant; linear regression lines displayed with CIs for each genotype subset; black dotted line depicts linear regression across all samples. All 5 CpGs in Fig 4, *B*, displayed associations with transcript levels of both the ANKRD55 and the upstream *IL6ST* genes, and CIT confirmed methylation-mediated regulation of these transcripts for cg21124310, cg10404427, cg23343972, and cg15431103 (Table II).

**Fig E4:**
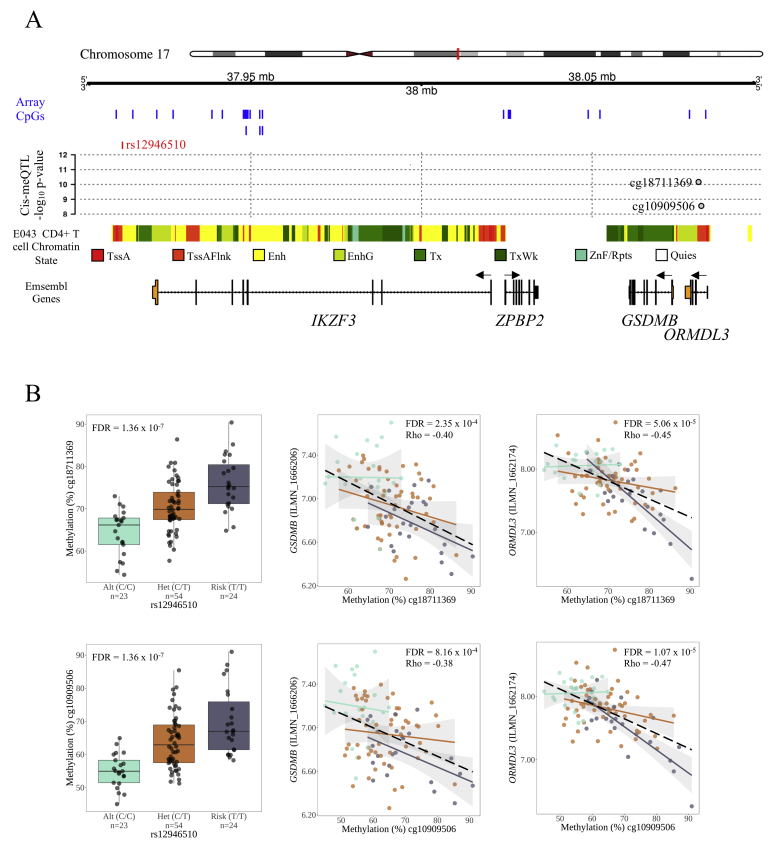


Given the potential of DNAm to influence gene expression, we incorporated paired, cell-type–specific transcriptome data to identify instances in which CpG methylation was simultaneously correlated with risk variants (meQTLs) and transcript levels of genes in *cis* (eQTMs). Focusing once more on RA, eQTMs within a ±500-kb window of *cis*-CpG sites at disease risk loci were mapped. Forty-two such instances were identified in CD4^+^ T cells, representing 20 CpGs and 8 unique genes (*ANKRD55*, *JAZF1*, *FCRL3*, *ORMDL3*, *IL6ST*, *GSDMB*, *C11orf10*, and *TAX1BP1*; see Table E15 in this article’s Online Repository at www.jacionline.org). In B cells, 65 associations encompassed 29 CpGs and 14 unique genes (*FAM167A*, *ORMDL3*, *GSDMB*, *IKZF3*, *FCRL3*, *XKR6*, *BLK*, *CCR6*, *SLC4A7*, *CDK12, IRF5, MSL1, NEIL2*, and *TXNDC11*; see Table E16 in this article’s Online Repository at www.jacionline.org). In both cell types, most associations were inverse, with increased methylation coinciding with decreased transcript levels (79% unique CpG-Gene pairs in CD4^+^ T cells, 66% B cells). Notably, DNAm at several CpG sites correlated with the expression of multiple genes, as in the examples of cg18711369 and cg10909506 on chromosome 17 (*ORMDL3* and *GSDMB* in CD4^+^ T cells; see Fig E4 in this article’s Online Repository at www.jacionline.org). To distinguish instances of probable DNAm-mediated genetic regulation of transcription from alternative regulatory models (Fig 4, *A*), we performed CIT (see this article’s Methods section in the Online Repository).^44^ As input, we included all triplets identified in our *cis*-meQTL and eQTM analyses (RA risk variant, CpG site, and transcript). In CD4^+^ T cells, CIT highlighted site-specific DNAm as a probable regulatory intermediate between risk variant-meQTLs and eQTMs at 5 risk loci (FDR < 0.05), implicating *ANKRD55*, *JAZF1*, *ORMDL3*, *FCRL3*, *IL6ST*, *C11orf10*, *TAX1BP1*, and *GSDMB* as genes with potential to confer perturbed immune function via this mechanism in RA. Notably, *FCRL3* was similarly implicated in B cells and although *ORMDL3*, *IKZF3*, and *CCR6* showed statistically significant methylation-mediated associations (*P* < .05), they were not robust to FDR correction in this cell type (Table II). Complete CIT results are available in Tables E17 and E18 in this article’s Online Repository at www.jacionline.org. We also performed a CIT analysis treating transcript levels as a mediator to identify potential instances of reverse causation, but no such effects were detected.

**Table II:** CIT identifies genes for which DNAm likely mediates RA genetic risk

Gene	CpG	Lead meQTL SNP	Bayes colocalization PP4∗	Locus	CIT *P* value	CIT Permutation FDR
**CD4**^**+**^**T cell**						
*ANKRD55*	cg21124310	rs6859219	1.00	5q11.2	1.11 × 10^−4^	7.06 × 10^−4^
	cg10404427	rs6859219	1.00		.0057	0.0044
	cg23343972	rs6859219	1.00		.0062	0.0069
	cg15431103	rs6859219	0.95		.0496	0.0319
*JAZF1*	cg07522171	rs2189966	0.97	7p15.1	3.97 × 10^−4^	0.0035
	cg11187739	rs4722758	0.99		.0035	0.0044
	cg16130019	rs917117	0.99		.0529	0.0319
*ORMDL3*	cg18711369	rs12946510	0.86	17q12	4.46 × 10^−4^	0.0035
	cg10909506	rs12946510	0.93		.0016	0.0044
*FCRL3*	cg17134153	rs2210913	0.99	1q23.1	.0027	0.0044
	cg01045635	rs2210913	0.99		.0120	0.0296
*IL6ST*	cg15431103	rs6859219	0.95	5q11.2	.0100	0.0296
	cg15667493	rs6859219	0.99		.0139	0.0296
	cg10404427	rs6859219	1.00		.0216	0.0305
	cg21124310	rs6859219	1.00		.0349	0.0305
	cg23343972	rs6859219	1.00		.0352	0.0305
*C11orf10*	cg16213375	rs61897793	1.00	11q12.2	.0163	0.0296
*TAX1BP1*	cg11187739	rs4722758	0.99	7p15.1	.0470	0.0305
*GSDMB*	cg18711369	rs12946510	0.86	17q12	.0277	0.0305
	cg10909506	rs12946510	0.93		.0448	0.0305
**B cell**						
*FCRL3*	cg19602479	rs2210913	0.99	1q23.1	4.69 × 10^−4^	0.0420
	cg01045635	rs7522061	0.97		5.49 × 10^−4^	0.0420
*CCR6*	cg15222091	rs3093025	0.98	6q27	.0101	0.0966
	cg19954286	rs3093025	0.98		.0258	0.1330
	cg05094429	rs3093025	0.96		.0347	0.1330
*IKZF3*	cg18691862	rs9903250	0.44	17q12	.0249	0.1330
*ORMDL3*	cg12749226	rs11557466	0.97	17q12	.0249	0.1330

For the top results in CD4^+^ T cells and B cells, we report the raw omnibus CIT *P* value, and FDR value calculated using 1000 permutations.^44^ Here, we report all instances of *P* < .05, highlighting those that satisfy the FDR 0.05 threshold. In instances whereby CIT implicates multiple Illumina probes for the same gene, we report the most statistically significant probe. Results from all CITs across both cell types are available in Tables E17 and E18.

∗ PP4 from the Bayesian colocalization analysis^38^ reports the previous probability that the associations with RA susceptibility and DNAm levels shared the same common genetic variant; only reported where PP4/PP3 > 5 (see this article’s Methods section in the Online Repository at www.jacionline.org).

**Fig E5:**
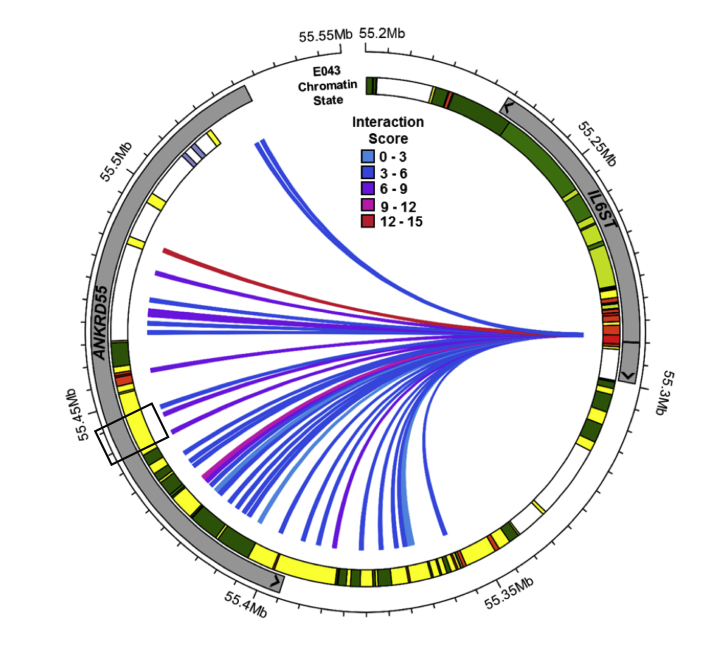


Intriguingly, not only do disease-associated SNPs frequently exhibit pleiotropic meQTL effects at these loci, but impacted CpGs may in turn regulate the expression of multiple putatively causal genes. In addition to the *GSDMB*/*ORMDL3* example given above, the rs6859219 SNP on chromosome 5 provides an interesting example, with associations between the risk variant and DNAm at 5 proximal CpG sites (Fig 4, *B* and *C*). Furthermore, methylation status at these sites correlates with the expression of both *IL6ST* and *ANKDR55* in CD4^+^ T cells (Fig 4, *C*), and CIT confirmed that DNAm at 4 of these sites (cg21124310, cg10404427, cg23343972, and cg15431103) likely regulates transcription of both *ANKRD55* and *IL6ST*. The risk variant and 2 of the CpG sites displaying the strongest association in CIT analysis (cg21124310 and cg10404427) map to an intronic enhancer at the *ANKRD55* gene. Using publicly available capture Hi-C data from CD4^+^ T cells,^49^ we noted that this intronic enhancer physically interacts with the *IL6ST* promoter, potentially explaining this coregulation in 3D space (see Fig E5 in this article’s Online Repository at www.jacionline.org).

### meQTL-mediated transcriptomic regulation highlights shared etiology at common IMD loci

**Fig 5:**
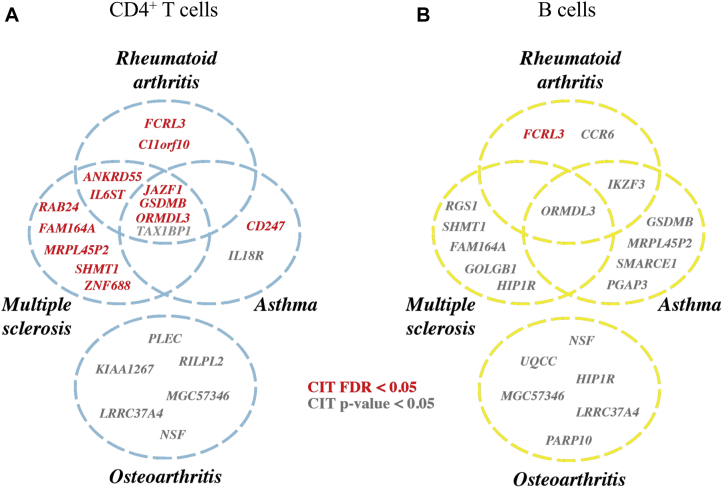
MeQTL-mediated transcriptomic regulation highlights shared etiology at common IMD loci. Venn diagrams showing the overlap of genes targeted by RA, MS, asthma, and OA risk loci for whom the genetic effect on expression in CD4^+^ T cells (**A**) and B cells (**B**) is mediated by DNAm according to CIT.

The above approach was applied to GWAS loci from studies of MS, asthma, and OA to highlight potential overlap in genetic etiology. Hence, across 55 independent (*r*^2^ > 0.8) MS-associated *cis*-meQTLs in CD4^+^ T cells, 36 unique eQTMs were mapped, comprising 16 unique genes (Table E15), with CIT indicating a putative DNAm-mediated regulatory model at 11 of these (Fig 5; Table E17). Although 15 unique genes were similarly highlighted in B cells (Table E16), no methylation-mediated relationships were identified (FDR < 0.05; Fig 5; Table E18). Across the 59 asthma-associated CD4^+^ T-cell *cis*-meQTLs, 27 eQTMs comprised 11 unique genes (Table E15), with DNAm a likely regulatory intermediary at 4 of these (DNAm-mediated gene expression was not confirmed at any of 30 similarly identified eQTMs in B cells; Fig 5; Table E18). Conversely, at OA risk loci, no DNAm-mediated relationships were revealed by CIT after controlling for FDR (Fig 5; Tables E17 and E18). A considerable degree of overlap in genes likely subject to DNAm-mediated regulation was observed across the 3 IMDs in CD4^+^ T (but not B) cells, highlighting cellular mechanisms of genetic risk that may be shared between disease phenotypes (Fig 5).

### Validation of *cis*-meQTL regulatory effect on causal gene expression at RA risk loci

**Fig 6:**
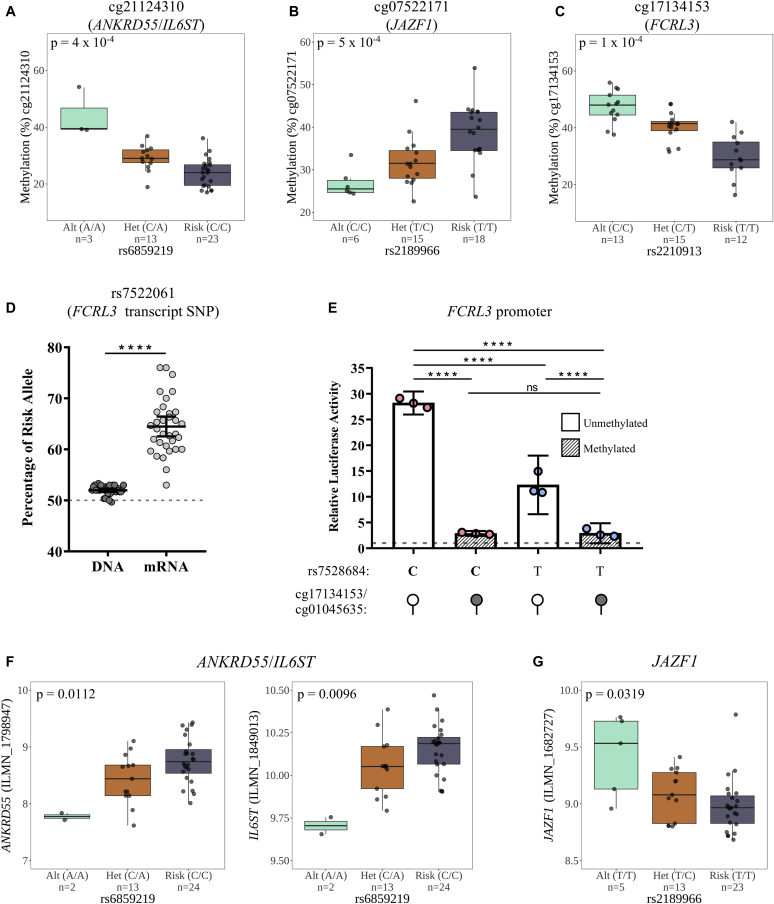
Validation of CD4^+^ T-cell meQTL and eQTL effects in independent patient cohorts. DNAm was quantified at (**A**) cg17134153 (*FCRL3* locus), (**B**) cg07522171 (*JAZF1* locus), and (**C**) cg21124310 (ANKRD55 locus) in CD4^+^ T cells using pyrosequencing. **D,***FCRL3* allelic expression imbalance analysis of individuals heterozygous for the transcript SNP rs7522061. **E,***In vitro* luciferase reporter analysis of the *FCRL3* promoter region containing the rs7528684 SNP and CpG sites cg17134153 and cg01045635 (see text and this article’s Methods section in the Online Repository). Mean ± 95% CI of 3 independent experiments are presented; ****P < .0001, Mann-Whitney *U* test. **F** and **G,** Validation of CD4^+^ T-cell eQTL effects for *ANKRD55* and *JAZF1*, respectively.

We validated *cis*-meQTL effects at 3 loci implicated by CIT (cg21124310/*ANKRD55*, cg07522171/*JAZF1*, and cg17134153/*FCRL3*) in an independent cohort of 39 patients with early arthritis using pyrosequencing (Fig 6, *A-C*). In addition, the presence of a proxy SNP (rs7522061) within the *FCRL3* transcript that is in high LD (*r*^2^ > 0.9 in Europeans, EUR) with the lead regulatory meQTL SNP (rs2210913; Table II) enabled us to perform allelic expression imbalance analysis to confirm the eQTL effect at this locus in CD4^+^ T cells. By quantifying relative allelic proportions at the proxy SNP within the genomic DNA and mRNA of a cohort of patients heterozygous at the regulatory SNP, we found the frequency of the risk variant (T at rs2210913, C at rs7522061) to be greatly enriched in mRNA (Fig 6, *D*). The lead meQTL variant (rs2210913) is in perfect LD (*r*^2^ > 0.99) with an SNP (rs7528684) that has been functionally validated as an eQTL variant associated with autoimmunity.^50^ We cloned this *FCRL3* promoter region containing rs7528684, as well as cg17134153 and cg01045635 (see Table II), into the pCpGl CpG-free vector harboring a luciferase reporter gene.^51^ Following transfection into Jurkat cells, transcriptional activity at the risk allele (C at rs7528684) was approximately 28-fold increased relative to the empty vector, and approximately 2.3-fold increased relative to the alternate (T) allele (Fig 6, *E*), confirming observations from allelic expression analysis. Crucially, *in vitro* methylation of the promoter region before transfection ablated this transcriptional activity regardless of the allele present, confirming that DNAm-associated transcriptional repression occurs downstream of the allelic effect (Fig 6, *E*), and providing clear experimental corroboration of previous statistical inference. The absence of an appropriate transcript SNP precludes allelic expression analysis at the other loci of interest; nonetheless, expression data for these samples were able to validate eQTL effects (Fig 6, *F* and *G*). Patients’ demographic data for the (me/e)QTL validation cohorts are detailed in Table E19 in this article’s Online Repository at www.jacionline.org.

### Multiple meQTLs acting in *trans* show evidence of phenotype-specific effects

**Fig E6:**
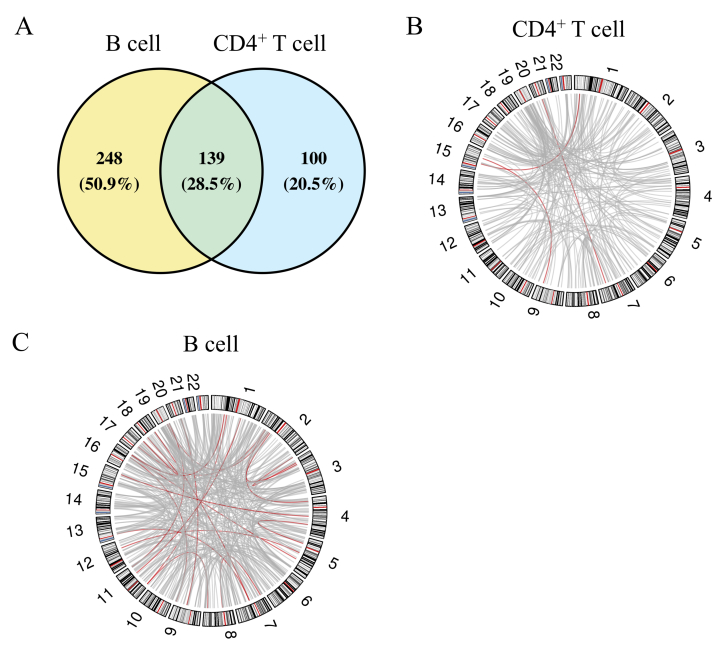


At the experiment-wide FDR threshold, 13,908 *trans* SNP-CpG associations (≥1 Mb) were identified in CD4^+^ T cells and 17,697 in B cells, of which 294 and 479 were independent after accounting for patterns of LD (Fig 1, *A*; see Tables E20 and E21 in this article’s Online Repository at www.jacionline.org), representing 239 and 387 individual CpGs, respectively. A total of 139 CpGs were regulated in *trans* in both cell types, again indicating a considerable degree of shared effects (see Fig E6, *A*, in this article’s Online Repository at www.jacionline.org). Most of the *trans* effects were interchromosomal (69.4% in CD4^+^ T cells, 71.6% in B cells). In contrast to the limited number of disease-specific effects highlighted in *cis*, interaction analysis identified a number of *trans* meQTL effects that differed significantly by disease phenotype: 3 independent examples were demonstrated in CD4^+^ T cells (Fig E6, *B*; Table E5) and 21 in B cells (Fig E6, *C*; Table E5)*.* However, no such effects localized to RA risk loci.

## Discussion

We present an integrated analysis of DNAm and paired transcriptomic data in genotyped CD4^+^ T and B cells of treatment-naive patients with early RA and disease controls. The study’s comprehensive molecular approach, directed at functionally relevant cellular compartments during disease induction, recognizes the relevance of biological context in an effort to unravel mechanisms of genetic risk. Indeed, the imperative to study genetic regulation at the level of defined cell populations is reinforced by our study, in which more than 30% *cis*-regulated CpGs were subset specific to either CD4^+^ T cells or B cells. As a whole, our findings provide an essential resource for the functional interpretation of GWAS and epigenome-wide association studies in RA, having further implications for IMD more generally.

Most significantly, we confirm the capacity of DNAm to act as an intermediary between disease-associated genetic variation and gene expression at a cellular level. Thirty-seven percent (41 of 112) of RA risk loci were found to harbor meQTL variants in CD4^+^ T and/or B cells in our study, and many of the affected CpGs in turn likely influenced transcriptional activity. In most cases, inverse association between DNAm and gene expression was consistent with a repressive function of methylation at gene promoters.^16^ The intronic RA SNP, rs6859219, at the chromosome 5q11.2 risk locus is striking for its robust association with autoantibody seronegative as well as seropositive RA.^52^ Here, the variant potentially regulates expression of 2 causal candidates in CD4^+^ T cells—*IL6ST*, which encodes the common cytokine receptor gp130, and *ANKRD55* whose function remains unknown—and appears to do so via both intergenic (cg23343972) and intronic (cg21124310, cg10404427) CpGs.

A further example of this phenomenon arises at the 17q12 locus, where the RA-associated variant mediates transcriptional repression of *ORMDL3*, likely doing so via increased methylation of at least 2 CpGs in CD4^+^ T cells. In addition to corroborating a mechanism of genetic risk previously proposed in asthma,^53^ the finding is intriguing given the recently characterized function of the gene product in suppressing IL-2 production by these cells.^54^ Our observations at this locus also illustrate the pleiotropic potential of site-specific methylation to mediate the expression of putatively causal genes. Accordingly, methylation at the cg18711369 position functions as an eQTM for *GSDMB* as well as *ORMDL3*. We also found convincing evidence for DNAm-mediated eQTL effects at the *JAZF1* gene, whose product is suggested to regulate proinflammatory signaling via p38 mitogen-activated protein kinase and/or nuclear factor kappa B,^55^ and the *FCRL3* gene. In relation to the latter, our experimental findings strongly support the directionality of observed associations, providing confidence in the CIT analytical approach generally—although the possibility that distinct variants in strong LD exert independent effects on DNAm and gene expression cannot be definitively excluded.^56^

Our study suggests that discrete genetic risk mechanisms span IMD phenotypes. For example, strong evidence is found for site-specific DNAm as an intermediary via which disease variants regulate *ORMDL3*, *GSDMB*, and *JAZF1* expression by CD4^+^ T cells in RA, MS, and asthma, and a similar mechanism in relation to *ANKRD55* and *IL6ST* is limited to RA and MS. In contrast, equivalent regulation of *FCRL3* expression appeared unique to RA in our study, albeit in *both* CD4^+^ *and* B cells. A concerted effort to understand the role of these putatively causal genes in lymphocyte pathobiology during the earliest stages of IMD (including RA) could pay dividends in the clinic, and should now be prioritized.

A tendency for the strongest meQTL regulatory effects to occur preferentially in *cis* in both cell types is congruent with previous findings from a range of tissues.^9^^,^^13^^,^^15^ Although increasing population sizes will be required to comprehensively map *trans*-meQTLs genome-wide, we do observe interchromosomal effects, many of which appear to be active in both cell types. Moreover, meQTL associations whose effects differ significantly between patients with RA and disease controls occurred predominantly in *trans.* Although preliminary, this may highlight regulatory effects that reflect the natural history of the inflammatory/immune pathology during early disease. Notably, instances of context-specific transcriptional regulation, dependent on disease state or exposure of cells to external stimuli, have been described.^26^^,^^27^ Examples of *cis*-regulation of DNAm that is specific to patient cohorts has been described in autoimmunity.^57^

In summary, we have demonstrated the central role of DNAm as an intermediary in regulating the lymphocyte transcriptome, highlighting molecular pathways through which genetic variation might confer dysregulated cellular function in RA and etiologically related IMDs. In so doing we highlight mechanisms of genetic risk in complex disease generally, prioritizing causal genes and tractable pathways for study as mediators of adaptive immune dysregulation. Emerging approaches for targeting site-specific DNAm *in vivo*^58^ may build on observations such as ours, raising the prospect of personalized therapeutic approaches to restore immune tolerance and effect cures for complex disease in the future.

**Key messages**

• DNAm associated with genetic variants at RA risk loci likely mediates causal gene expression in CD4^+^ and B lymphocytes.
• This mechanism is common to IMDs with overlapping genetic architectures.

## Supplementary data

Online Repository

Tables E1-E21
