# Supplementary material for: Lymphocyte DNA methylation mediates genetic risk at shared immune-mediated disease loci
Source: J Allergy Clin Immunol. 2020 May;145(5):1438–51. doi: 10.1016/j.jaci.2019.12.910 (PMC7201180; doi:10.1016/j.jaci.2019.12.910)
Supplement: Online Repository [file mmc1.docx]

**Lymphocyte DNA methylation mediates genetic risk at shared immune mediated disease loci.**

**Supplementary Methods.**

Alexander D Clark, Nisha Nair, Amy E Anderson, Nishanthi Thalayasingam, Najib Naamane, Andrew J Skelton, Julie Diboll, Anne Barton, Stephen Eyre, John D Isaacs, Arthur G Pratt* and Louise N Reynard.

*Corresponding author; [arthur.pratt@ncl.ac.uk](mailto:arthur.pratt@ncl.ac.uk)

**Supplementary Methods.**

**Lymphocyte-specific nucleic acid isolation from patients.** Patients with suspected inflammatory arthritis were enrolled from the Newcastle Early Arthritis Cohort (NEAC) prior to commencement of immunomodulatory therapy, as described in detail elsewhere^30^. All subjects were of Northern European ancestry (self-reported and confirmed by genotype analysis as part of quality control in PLINK). Initial diagnoses were validated at follow-up visits over a median 20 month period (range 13-25), with reference to 2010 ACR/EULAR classification criteria for RA^31^. The disease control population was selected to be equivalent to the RA population in respect of age, sex and acute phase markers (Erythrocyte sedimentation rate; ESR, and C-reactive protein; CRP) at the time of blood draw. The study was approved by the Newcastle and North Tyneside Regional Ethics Committee, and all participants gave written informed consent. CD4^+^ T cells and CD19^+^ B cells were isolated from the peripheral blood samples using a magnetic bead-based positive selection as we have previously described^8^, with isolation of paired, high-integrity RNA and DNA using the AllPrep DNA/RNA Mini Kit (Qiagen, UK).

**Genotyping.** Genotyping was carried out using an Illumina Human CoreExome‐24 version 1‐0 array (Illumina). Samples and SNPs with a call rate of <98% were removed. In addition, SNPs with a minor allele frequency of <0.01 or Illumina GenomeStudio cluster separation of <0.4 were excluded from further analysis. Data were pre‐phased with SHAPEIT2^32^ and imputed to the 1000 Genomes Phase 3 reference panel using IMPUTE2^33^. Imputed SNPs with INFO scores of <0.8 were excluded. Potential duplicate samples and relatedness between individuals were identified using identity-by-descent analysis in PLINK^E59^. If pairs of individuals with a PI_HAT >0.2 were identified, the sample with the lowest call rate was excluded, if any. QTL analysis was limited to SNPs for which there were ≥3 individuals represented per genotype or, in the absence of minor allele homozygotes, ≥8 heterozygous individuals.

**DNA Methylation Quantification.** 400ng DNA was bisulphite-converted with the EZ-96 DNA Methylation kit (Zymo Research, Orange, CA), and DNAm quantified using the Illumina Infinium MethylationEPIC BeadChip according to manufacturer’s recommendations. CD4^+^ T- and B cell data were independently pre-processed using the minfi package in R^E60^. Failed probes whose detection p-values were >0.01 in >10% samples were excluded after functional normalisation^34^, and additional probes removed due to (i) cross-hybridization^E61-E64^, (ii) mapping to sex chromosomes, or (iii) presence of a SNP (MAF>0.05) at the interrogated CpG, probe sequence or single base extension site; cis-meQTLs were defined as those occurring over a distance of < 1Mb, with all other associations considered as trans-meQTLs. Potential sources of technological or biological confounding were estimated using surrogate variable analysis (SVA)^35^. No adjustment variables were included in the null model to allow all confounding variables to be estimated from the data, and diagnosis (RA/non-RA) was included in the full model to allow such potential effects to be preserved for downstream interaction analyses. A total of 14 and 13 surrogate variables were identified in CD4^+^ T cell and B cell data respectively. For ease of reference, methylation data are presented as *β*-values converted to a percentage (range 0 - 100), though statistical analyses are performed using M-values due to their homoscedastic nature. For the purpose of plotting DNAm and gene expression data (see later section), the removeBatchEffects function in the Limma package^39^ was applied to adjust data for covariates.

**Methylation quantitative trait locus analysis.** Associations between genotype and DNAm levels were identified by fitting additive linear models for SNP-CpG pairs using the MatrixEQTL package^36^. *Cis*-meQTL effects were defined where the SNP-CpG association occurred over a distance of <1Mb, with all other associations considered to be acting in *trans*. Disease diagnosis (RA/non-RA) and surrogate variables were included as covariates in the model. Analysis was limited to SNPs for which there were ≥3 individuals per genotype or, in the absence of minor allele homozygotes, ≥8 heterozygous individuals. Benjamini-Hochberg corrected FDR values were calculated separately for cis and trans associations, and values <0.01 (cis) and <1 x 10^-5^ (trans) across the total number of CpG-SNP pairs tested were considered statistically significant^E65^. Disease-specific meQTL effects were sought by performing the above analyses with the inclusion of a *genotype × diagnosis* interaction term in MatrixEQTL. This analysis was limited to SNPs for which each genotype was represented by ≥3 samples in each comparator group (RA or non-RA), and an FDR of < 0.05 (cis) or < 0.001 (trans) considered statistically significant for discovery purposes. To further mitigate against potential false-positive results in our interaction analysis, CpGs at which 90^th^ and 10^th^ percentile methylation values differed by <5% were excluded. Following the mapping of meQTLs, independent effects were distinguished from tagging SNPs in LD by clumping in PLINK^E59^. SNPs were clumped at a physical distance of 250kb, using an r^2^ threshold of 0.001, maintaining the SNP with the lowest p-value.

**Multivariate adaptive shrinkage analysis**. Significant meQTLs in at least one cell type were additionally analysed using the multivariate adaptive shrinkage (MASH) method^47^, as implemented in the mashr R package (https://github.com/stephenslab/mashr), to provide a more accurate assessment of effect sharing and specificity among T- and B-cells. MASH is an Empirical Bayes method that first learns patterns of sharing, sparsity and correlations present among the results of a condition-by-condition genomic analysis and then uses that information to improve effect estimates and their measures of significance, thereby increasing the power to detect effect sharing across multiple conditions**^47^**. We first generated data-driven and canonical covariance matrices, representing the possible patterns of effects present in our condition-by-condition meQTL analysis results, from the summary statistics of the 107,230 significant (FDR < 0.01) and 1M randomly-selected SNP-CpG associations respectively. A MASH model that incorporates these covariance matrices was then fitted to the random meQTLs and used to estimate posterior statistics for the significant meQTLs. meQTLs with a local false sign rate (lfsr) less than 0.05 were considered statistically significant^47^.

**Co-localisation analysis**. We sought to integrate our meQTL analysis results with previous RA GWAS findings. GWAS trait-associated SNPs were extracted from publicly available GWAS catalog datasets^37^, restricting our search to SNPs that passed genome-wide significance (p < 5 x 10^-8^). Risk loci were subsequently defined as all SNPs in high LD (r^2^ > 0.8 based on the 1000 Genomes Phase 3 data in EUR Populations) with these lead risk variants. We then identified the overlaps between significant meQTLs and these risk loci to define risk-associated meQTLs. Subsequent to this, in order to provide additional support for co-localisation, we applied the Bayesian co-localisation method implemented in the coloc R package^38^ to statistically assess the probability that an observed overlap is due to the presence of a single causal variant common to both DNA methylation and RA risk. For each co-localisation test, meQTL and GWAS summary statistics of all the SNPs within 1Mb of the meQTL CpG were inputted to the coloc.abf function which returns posterior probabilities (PPs) for the presence of two independent (PP3) or one shared (PP4) causal variant(s) affecting the tested traits. A PP4 > 0.75 and a PP4/PP3 > 5 were required for co-localisation significance.

**Gene Expression Quantification.** Generation of contemporaneous, genome-wide CD4+ T and B cell transcriptomic data from the same cohort of subjects using Illumina HumanHT-12 v4 BeadChip has been described^8^. Here, probes whose detection p-value was >0.05 in over 25% of samples were excluded prior to background correction and quantile normalised using the Limma package^48^, following which additional probes were removed where they mapped to (i) sex chromosomes, (ii) repeat or intronic/intergenic regions or (iii) unmapped regions. Batch effects related to technical variability were accounted for using the SVA package in a manner identical to that described for DNAm data above^35^, capturing 15 & 12 surrogate variables in CD4+ T cell and B cell data respectively.

**Chromatin state and transcription factor binding enrichment analysis.** Cis-CpG sites were mapped to cell-specific chromatin states from the Roadmap 15-state model, determined by applying a Hidden Markov Model (HMM) to data for five histone modifications (H3K4me3, H3K4me1, H3K36me3, H3K27me3, H3K9me3)^40^. Specifically, cis-CpGs associated with meQTLs in CD4^+^ T cell and B cell datasets were mapped to chromatin states of primary T helper cells (E043) and primary B cells (E032) from peripheral blood, respectively. To facilitate interpretation, the 15 annotated states were collapsed into five intuitive annotations; 1) transcription start site (TssA/TssBiv), 2) flanking a transcription start site (TssAFlnk/BivFlnk), 3) Enhancers (EnhG, Enh, EnhBiv), 4) Transcribed (TxFlnk, Tx, TxWk), and 5) Repressed (ZNF/Repeats, Het, ReprPC, ReprPCWk, Quies). We also overlapped CpG sites with transcription factor binding site (TFBS) data leveraged from the ENCODE ChIP-seq datasets^41, 42^. All significant enrichments were assessed using two-way Fisher’s exact test and, in the case of TFBS, Bonferroni-corrected p-values calculated to adjust for all TFs tested per trait in each cell type. To explore whether CpGs regulated in cis are functionally enriched in specific biological pathways, ontology analysis was performed using a modified hypergeometric test within the MissMethyl package^43, E66^, accounting for probe bias as described^E67^. Cis-CpGs associated with non-risk loci were used as background for all enrichment analyses.

**Expression Quantitative Trait Methylations.** At meQTL-associated cis-CpG sites we applied Spearman’s rho to identify expression quantitative trait methylations (eQTMs), where CpG methylation is associated with transcript levels of genes within a ±500kb window. To negate the impact of confounding variables, correlations were performed on DNAm and expression residuals, after adjusting for diagnosis (RA/non-RA) and surrogate variables (see above). Correlations were selected at an FDR <0.01, calculated based on the number of transcripts tested per cis-CpG.

**Causal Inference Testing** To infer directionality of effects at SNP-CpG-Transcript triplets, we employed the causal inference test (CIT)^44^. This calculates the likelihood than an observed association between a genomic locus (L) and the phenotypic trait of interest (P) occurs via a potential mediator (M). CIT performs four statistical tests that must be satisfied to conclude causality; 1) L is associated with M, 2) L is associated with P, 3) L is associated with M after conditioning on P, and 4) L is independent of P after conditioning on M. In the model, we included triplets at RA risk loci that demonstrated both *cis*-meQTL and *cis*-eQTM effects, treating DNAm as a mediator and transcript levels the observed outcome phenotype. We determined FDR values by performing 1000 permutations of the data, considering values <0.05 to infer mediation at the molecular level.

**Bisulphite Pyrosequencing**

250ng CD4^+^ T cell patient DNA was bisulphite-converted DNA (EZ DNA methylation kit ,Zymo research) was amplified in a 20µl reaction ((1x TITANIUM Taq Buffer, Clontech, Saint-Germain-en-Laye, France), 250nM forward and reverse primers (*Supplementary Table 1*), 0.2mM dNTPs, 1x TITANIUM Taq DNA Polymerase (Clontech), 1µl bisulphite-converted DNA)). Amplification was carried out at 95°C for 1 minute, followed by 40 cycles of 95°C for 15 seconds, 63°C (cg07522171 & cg21124310) or 68°C (cg17134153) for 1 minute, and 68°C for 1 minute, with a final extension at 68°C for 5 minutes.

Following the isolation of single-stranded DNA using the Pyromark Q24 vacuum workstation (Qiagen), pyrosequencing was performed on the Pyromark Q24 instrument with Pyromark Gold Q96 reagents (Qiagen) and site-specific sequencing primers (0.3µM, *Supplementary Table 1*).

**Allelic Expression Imbalance**

400ng CD4^+^ T cell RNA was reverse transcribed using Superscript II Reverse Transcriptase (ThermoFisher) in a 20µl final reaction volume following the manufacturer’s protocol. The region containing the variant of interest was amplified by PCR in both the genomic DNA (gDNA) and complimentary DNA (cDNA). 20ng sample DNA was prepared in a 20µl reaction containing 1x PCR Gold Buffer (ThermoFisher), 1.2mM MgCl_2_, 0.5µM each of forward and reverse primer (*Supplementary Table 1*), 0.25mM dNTPs, 0.5U AmpliTaq Gold DNA Polymerase (ThermoFisher). DNA was then amplified at 95°C for 10 minutes, 40 cycles of 95°C for 15 seconds, 57°C for 30 seconds, and 72°C for 30 seconds, with final extension at 72°C for 5 minutes.

Allele proportions in the gDNA and cDNA were quantified as before by pyrosequencing using the Pyromark Q24 (see bisulphite pyrosequencing) with a site-specific sequencing primer (0.3µM, *Supplementary Table 1*).

**Luciferase Reporter Assay** A 974bp region (chr1:157,670,120 – 157,671,093; hg19) encompassing the *FCRL3* promoter, and harbouring three SNPs (rs7528684, rs11264799, and rs945635), as well as cg17134153 and cg01045635, was PCR amplified from template human genomic DNA heterozygous at rs7528684 (*Supplementary Table 1* for primer sequences). Amplified regions were cloned into the pCpGL-basic vector using SpeI and NcoI restriction enzymes (New England Biolabs). ChemiComp GT115 E.coli cells (Invivogen) were transformed with the construct and subsequently selected on LB agar plates supplemented with Zeocin (30µg/ml). Sanger sequencing (Source Bioscience, Nottingham, UK) confirmed ligation and correct orientation of the insert, and enabled identification of clones that differed at two positions (rs7528684 and rs945635, which are in perfect LD). 30µg of each construct, as well as the empty vector, was purified (PureYield™ Plasmid Maxiprep System, Promega) and either *in vitro* methylated (MSssI enzyme, 40U) or mock-methylated (H_2_O) in the presence of S-adenosyl methionine (SAM, 0.3mM), after which constructs were purified using the Wizard® SV Gel and PCR Clean-Up System (Promega). 2.5 x 10^5^ Jurkat cells (Clone E6-1) were co-transfected with 1.5µg of either methylated or mock-methylated DNA (either C/T allele at rs7528684 or empty vector), as well as 30ng plasmid containing *Renilla* luciferase reporter gene, using the Neon Electroporation Kit (1400V, 10ms, 3 pulses; Invitrogen). Following a 24-hour incubation period, luciferase activity was quantified using the Dual Luciferase Reporter Assay system (Promega), with firefly luciferase activity normalised to *Renilla* luciferase values, and relative luciferase activity normalised to the appropriate methylated/mock-methylated empty vector. Each condition was performed in triplicate.

**Statistics**

Statistical analyses were performed in R version 3.4.4 and GraphPad Prism 7. A p-value threshold of <0.05 was considered significant unless otherwise stated.

**Supplementary References**

E59. Purcell S, Neale B, Todd-Brown K, Thomas L, Ferreira MAR, Bender D, et al. PLINK: A tool set for whole-genome association and population-based linkage analyses. American Journal of Human Genetics 2007; 81:559-75.

E60. Aryee MJ, Jaffe AE, Corrada-Bravo H, Ladd-Acosta C, Feinberg AP, Hansen KD, et al. Minfi: a flexible and comprehensive Bioconductor package for the analysis of Infinium DNA methylation microarrays. Bioinformatics 2014; 30:1363-9.

E61. Benton MC, Johnstone A, Eccles D, Harmon B, Hayes MT, Lea RA, et al. An analysis of DNA methylation in human adipose tissue reveals differential modification of obesity genes before and after gastric bypass and weight loss. Genome Biology 2015; 16:21.

E62. Chen YA, Lemire M, Choufani S, Butcher DT, Grafodatskaya D, Zanke BW, et al. Discovery of cross-reactive probes and polymorphic CpGs in the Illumina Infinium HumanMethylation450 microarray. Epigenetics 2013; 8:203-9.

E63. Pidsley R, Zotenko E, Peters TJ, Lawrence MG, Risbridger GP, Molloy P, et al. Critical evaluation of the Illumina MethylationEPIC BeadChip microarray for whole-genome DNA methylation profiling. Genome Biology 2016; 17:17.

E64. McCartney DL, Walker RM, Morris SW, McIntosh AM, Porteous DJ, Evans KL. Identification of polymorphic and off-target probe binding sites on the Illumina Infinium MethylationEPIC BeadChip. Genomics Data 2016; 9:22-4.

E65. Benjamini Y, Hochberg Y. CONTROLLING THE FALSE DISCOVERY RATE - A PRACTICAL AND POWERFUL APPROACH TO MULTIPLE TESTING. Journal of the Royal Statistical Society Series B-Statistical Methodology 1995; 57:289-300.

E66. Young MD, Wakefield MJ, Smyth GK, Oshlack A. Gene ontology analysis for RNA-seq: accounting for selection bias. Genome Biology 2010; 11:12.

E67. Geeleher P, Hartnett L, Egan LJ, Golden A, Ali RAR, Seoighe C. Gene-set analysis is severely biased when applied to genome-wide methylation data. Bioinformatics 2013; 29:1851-7.

**From Supplementary Tables**

E68. Stahl EA, Raychaudhuri S, Remmers EF, Xie G, Eyre S, Thomson BP, et al. Genome-wide association study meta-analysis identifies seven new rheumatoid arthritis risk loci. Nature Genetics 2010; 42:508-U56.

E69. Plenge RM, Cotsapas C, Davies L, Price AL, Bakker PIW, Maller J, et al. Two independent alleles at 6q23 associated with risk of rheumatoid arthritis. Nature Genetics 2007; 39:1477-82.

E70. Gregersen PK, Amos CI, Lee AT, Lu Y, Remmers EF, Kastner DL, et al. REL, encoding a member of the NF-kappa B family of transcription factors, is a newly defined risk locus for rheumatoid arthritis. Nature Genetics 2009; 41:820-U77.

E71. Saad MN, Mabrouk MS, Eldeib AM, Shaker OG. Studying the effects of haplotype partitioning methods on the RA-associated genomic results from the North American Rheumatoid Arthritis Consortium (NARAC) dataset. Journal of Advanced Research 2019; 18:113-26.

E72. Okada Y, Terao C, Ikari K, Kochi Y, Ohmura K, Suzuki A, et al. Meta-analysis identifies nine new loci associated with rheumatoid arthritis in the Japanese population. Nature Genetics 2012; 44:511-+.

E73. Orozco G, Viatte S, Bowes J, Martin P, Wilson AG, Morgan AW, et al. Novel Rheumatoid Arthritis Susceptibility Locus at 22q12 Identified in an Extended UK Genome-Wide Association Study. Arthritis & Rheumatology 2014; 66:24-30.

**Online Repository Figure Legends.**

**Figure E1**. Overview of the study design and key findings.

**Figure E2**. Overlap of CpG sites subject to cis-meQTL in CD4^+^ T cells and B cells. **A**. Overlap of cis-meQTL CpGs between CD4^+^ T cells and B cells identified in a comparative analysis. **B**. Overlap of cis-meQTL CpGs between CD4^+^ T cells and B cells identified using a multivariate adaptive shrinkage analysis (MASH) approach (see Supplementary methods for details). **C**. Comparison of direction and effect size (β coefficient) of overlapping CD4^+^ T cell (X axis) and B cell (Y axis) cis-meQTLs identified in **A**. Those displaying opposing allelic effects on DNAm levels between the two cell types are highlighted in red.

**Figure E3**. cis-meQTL plots illustrating a disease x genotype interaction effect at rs13145446 genotype on DNA methylation at cg23683081 in B cells. The G allele is associated with increased methylation in non-RA individuals, but decreased methylation in RA patients. The interaction p-value denotes the significance of the genotype x diagnosis interaction effect in the linear model; individual p-values for meQTL associations in the RA and non-RA cohorts are also shown.

**Figure E4**. **A**. The RA risk variant rs12946510 (red line) on chromosome 17 is associated with DNAm at one intronic (cg18711369) and one exonic (cg10909506) CpG site (grey circles), mapping to CD4^+^ T cell enhancer regions (lime green and yellow). Blue lines indicate the position of all CpG sites included in the meQTL analysis. **B**. meQTL (left) and *GSDMB* (middle) and *ORMDL3* (right) methylation-expression plots for cg18711369 (top) and cg10909506 (bottom); regression lines depicted as for Figure 4 in main manuscript. Methylation at both of these CpG sites mediated expression of *ORMDL3* and *GSDMB* (see also Table 2).

**Figure E5**. Capture Hi-C data from CD4^+^ T cells (see Reference 49) revealed an interaction between the intronic enhancer at *ANKRD55* and the promoter of *IL6ST*. Black box indicates the intronic enhancer harbouring rs6859219, cg10404427, and cg21124310.

**Figure E6**. Disease-specific *trans*-meQTL effects. **A**. Overlap of CpG sites subject to trans-meQTL effects in CD4^+^ T cells and B cells. **B**. Circos plot illustrating all inter-chromosomal trans-meQTLs in CD4^+^ T cells. Highlighted in red are *trans* effects for which interaction analysis indicates phenotype (RA/non-RA) significantly impacts the observed trans-meQTL effect. **C**. Circos plot of *trans*-meQTLs in B cells, again highlighting those subject to significant interaction effects in red.
